# Acute kidney injury following percutaneous mechanical thrombectomy of subclavian artery stent graft thrombosis: a case report

**DOI:** 10.1186/s42155-020-00119-0

**Published:** 2020-06-01

**Authors:** Tajana Turk, Darko Blaskovic, Ranko Smiljanic, Vinko Vidjak

**Affiliations:** 1 Department of Diagnostic and Interventional Radiology, University Hospital Osijek, Huttlerova 4, 31000 Osijek, Croatia; 2 Faculty of Medicine, J.J.Strossmayer University of Osijek, Osijek, Croatia, Huttlerova 4, 31000 Osijek, Croatia; 3 Department of Diagnostic and Interventional Radiology, University Hospital Merkur, Zajceva ul.19, 10000 Zagreb, Croatia; 4 School of Medicine, University of Zagreb, Salata 3, 10000 Zagreb, Croatia

**Keywords:** Acute kidney injury, Aneurysm, Subclavian artery, Thrombectomy, Thrombosis

## Abstract

**Background:**

Percutaneous mechanical thrombectomy (PMT) is a well-established technique for treatment of acute arterial and venous thrombosis which inevitably leads to intravascular erythrocyte hemolysis, resulting in hemoglobinuria.

**Case presentation:**

We present a case of 66-year-old Caucasian female with subclavian artery aneurysm causing distal embolization and hand ischemia. The aneurysm was treated with stent graft, but with a subsequent graft thrombosis 3 months later. After graft recanalisation, AngioJet PMT was performed which resulted in dialysis-requiring acute kidney injury.

**Conslusion:**

Only several cases of acute kidney injury following AngioJet PMT have been published in literature. To our knowledge, this is the first reported case of dialysis-requiring AKI after PMT for peripheral arterial thrombosis. Until there is sufficient evidence and recommendation on preventing AKI in this setting, we believe that by being aware of the risk and by monitoring of patient, one might minimize the damage in case it occurs.

## Background

Percutaneous mechanical thrombectomy (PMT) is a well-established technique for treatment of acute thrombosis. The AngioJet system (Boston Scientific, USA) uses high-velocity saline jets to fragment and aspirate thrombus. This inevitably leads to intravascular hemolysis, resulting in hemoglobinuria. Cell-free hemoglobin can cause renal tubular damage by oxidative reactions which trigger a heme-toxicity response. Only several cases of acute kidney injury (AKI) following AngioJet PMT have been published. This is the first reported case of dialysis-requiring AKI after PMT for peripheral arterial thrombosis.

## Case presentation

A 66-year-old Caucasian female was referred for evaluation of subclavian artery aneurysm causing distal embolization and hand ischemia.

**Fig. 1:**
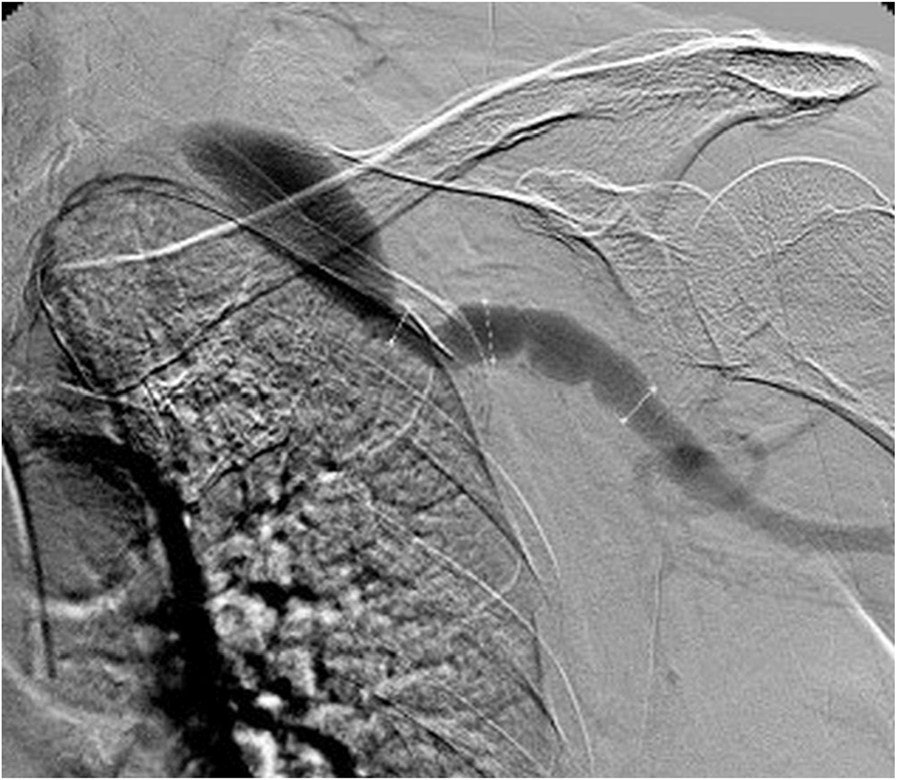
Digital subtraction angiography via transbrachial approach showing fusiform aneurysm of left subclavian artery extending into left axillary artery

Her medical history was notable of hypertension and hyperlipidemia without notice of renal insufficiency. She was an ex-smoker, had myocardial infarction 20 years earlier, bilateral femoropopliteal bypasses (15 and 8 years earlier) and operative repair of right common femoral artery pseudoaneurysm 4 years earlier. A computed tomography angiography showed aneurysm of extrathoracic segment of left subclavian artery. A multidisciplinary team (MDT) decided upon endovascular treatment. Digital subtraction angiography (DSA) confirmed 9 cm long fusiform aneurysm of left subclavian artery extending into left axillary artery, with a maximal diameter of 13 mm (Fig. 1). Since transfemoral approach was to be avoided due to previous bilateral groin surgeries, a 10 × 150 mm stentgraft (Viabahn, Gore, USA) was placed via a left brachial artery approach. Tubular graft was chosen based on diameter of non-diseased artery with a 1 mm difference in diameter of proximal and distal landing zone. During the procedure, the patient received 5000 IU of Heparin. Postprocedural course was uneventful, the patient was on low molecular Heparin for three days and was discharged with recommendation of dual antiplatelet therapy (Clopidogrel 75 mg/day, Aspirin 100 mg/day) for six months. The patient had a follow-up at GP office which showed good clinical outcome, as well as duplex sonography one month after the procedure which showed good graft patency.

**Fig. 2:**
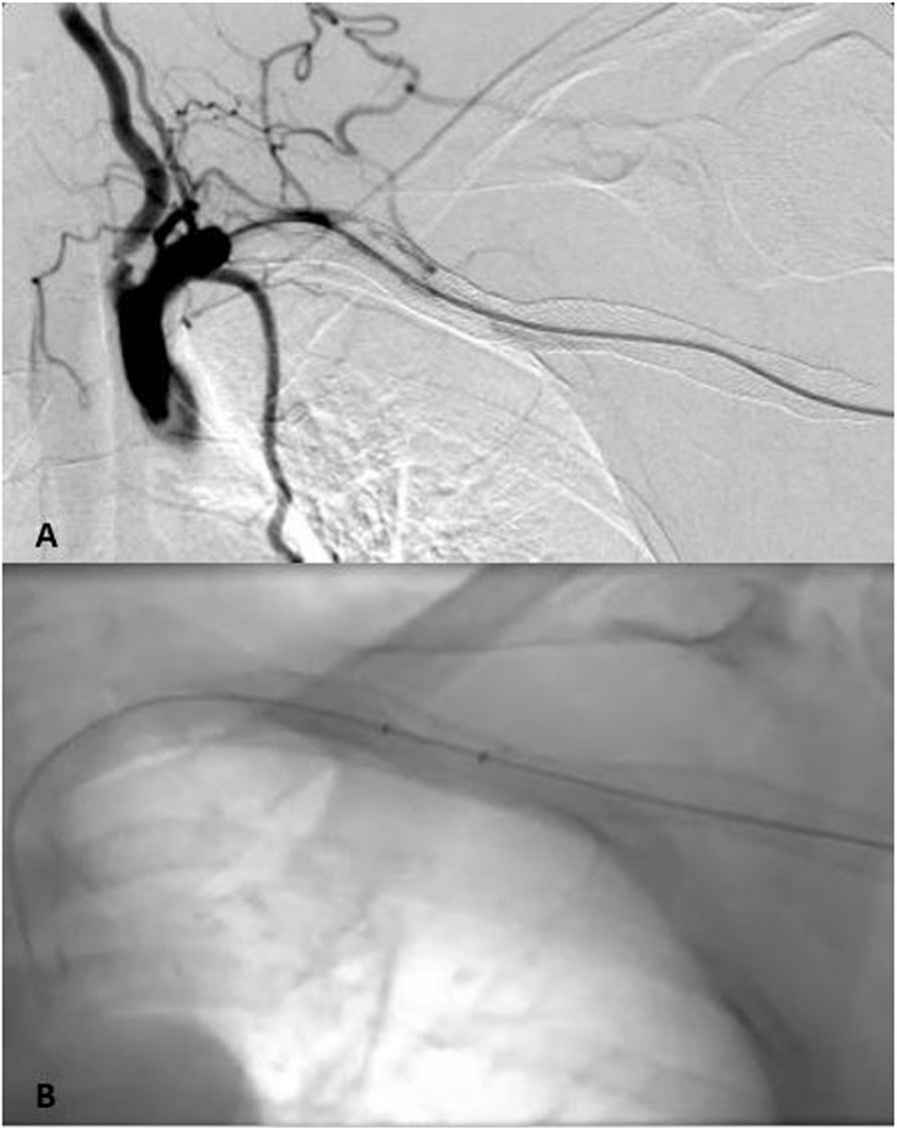
**a** Successful graft recanalisation by hydrophilic guidewire and vertebral catheter; **b** Pharmacomechanical thrombectomy performed with the AngioJet Thrombectomy System

**Fig. 3:**
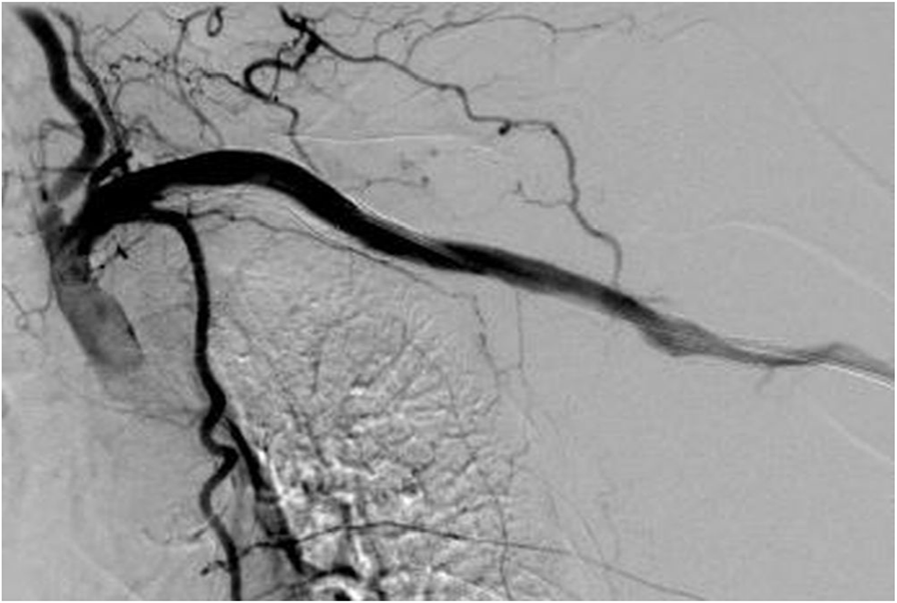
Postinterventional angiography showing good graft patency

**Fig. 4:**
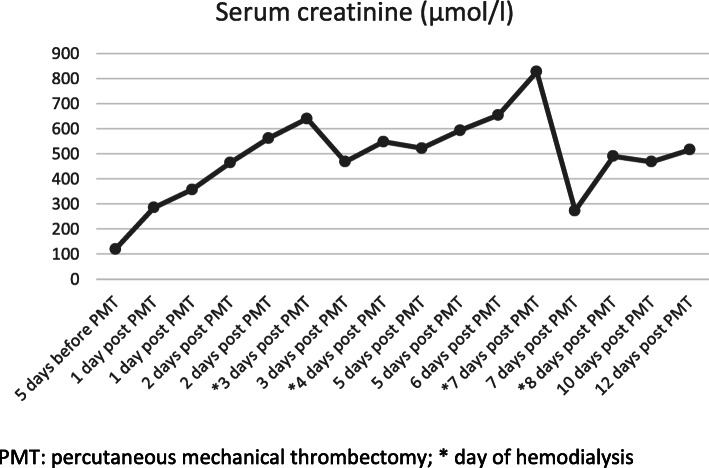
Patient’s serum creatinine levels (μmol/l) before and after percutaneous mechanical thrombectomy

The patient returned 3 months later with a sudden onset of left arm pain and pallor. Ultrasound showed complete graft thrombosis. The patient confirmed that she was not taking the prescribed antiagregation therapy regulary. Angiography via left transbrachial approach confirmed stent graft occlusion. After graft recanalisation, AngioJet PMT was performed after thrombus lacing with 10 mg of t-PA, with a total aspiration time of 180 s (Fig. 2). Postinterventional angiography showed good graft patency (Fig. 3). A total amount of 60 ml of nonionic contrast media was used. At the end of the procedure, the patient became agitated, with blood pressure 230/120 mmHg, chest pain and vomiting, they were transferred to intensive unit where myocardial infarction was excluded. The patient became oliguric with elevated serum creatinine of 285 μmol/l (prepocedural serum creatinine was 120 μmol/l, eGFR 41 ml/min/1.73m^2^). Blood tests at 48 h postprocedure showed significant drop in hematocrit (0,433 L/L to 0,284 L/L) as a marker of intravascular hemolysis as well as elevated LDH (1521 U/l), AST (303 U/L) and ALT (165 U/L). Due to AKI and continuous rise in serum creatinine (Fig. 4), urgent renal replacement therapy was initiated. The peak value of serum creatinine was 827 μmol/l on 7th postprocedural day. After 4 session of haemodialysis within 2 weeks, the patients’ renal function entered a polyuric phase and subsequent recovery without further need for hemodyalisis. Renal duplex sonography showed normal renal vasculature. Urine tests showed no signs of nephritis. No co-medication or concomitant disease was identified as a probable cause of renal failure. The pretreatment level of pottasium was 4,7 mmol/L, which was within the normal range. On final discharge, the patient was anticoagulated with Clopidogrel 75 mg/day. During 12-months follow-up, the left subclavian and axillary artery remained patent. Unfortunately, the patient died 12 months after PMT due to traumatic incident.

## Discussion

Acute arterial graft thrombosis resulting in acute limb ischemia is a limb threatening condition with a need for emergent treatment. Apart from open surgery, endovascular treatment options are available – catheter directed thrombolysis (CDT), percutaneous mechanical thrombectomy (PMT) and acoustic pulse thrombolysis. Since only 5% of limb ischemia affect the upper limb, literature upon the treatment is scarce, although CDT for upper limb ischemia can be effective in over 60% of patients, the most feared of complication is bleeding, with major hemorrhage incidence of up to 14% (Schrijver et al. 2016). The potential benefits of PMT are shorter procedure duration, cost-reduction, less volume of contrast media, possibility of delivering bolus of thrombolytic, decline in bleeding and overall complications.

AngioJet PMT system uses the Venturi-Bernoulli effect to macerate and aspirate thrombus. Although postprocedural hemolysis and hemoglobinuria are common, developing severe AKI requiring renal replacement therapy is an infrequent complication. Four of these cases occurred after PMT for venous thrombosis (Arslan et al. 2007; Bedi, and Daniel, and Reichman, Aaron, and Chou, Shyan-Yih, and Reiser, Ira 2018; Mathews et al. 2011; Sebastian et al. 2018) and one following PMT for pulmonary embolism (Dukkipati et al. 2009). We found no published cases reporting AKI after AngioJet PMT for peripheral arterial thrombosis.

The registry of AngioJet Use in the Peripheral Vascular System (PEARL II) (Registry of AngioJet Use in the Peripheral Vascular System 2018) reports renal failure rate of 0,47% in patients treated for limb ischemia. The use of AngioJet was found to be an independent risk factor for developing AKI, increasing the odds by a factor of eight (Escobar et al. 2017). Retrospective study on 145 patients with thrombosis, also reported higher incidence of AKI in PMT group, proposing more vigilant renal protective measures in post-PMT patients (Morrow et al. 2017).

We believe that, in our patient, previously unrecognized chronic kidney disease, might have played a role in developing AKI. Although intravenous iodinated contrast is associated with contrast induced nephropathy, it is not shown to be an independent risk factor for developing AKI. Our patient had received approximately the same volume of ionated contrast during first endovascular procedure, 3 months before PMT, without deterioration in kidney function tests. However, during the second procedure, hemolysis was an additional factor for developing AKI. Due to acute graft thrombosis, MDT decided that the risk of potential complications associated with PMT was acceptable.

Although there is no consensus upon which eGFR would be a cut-off value for administration of intravenous contrast, studies suggest that there is no significant risk for AKI in patients with eGFR> 30 ml/min/1.73m^2^ (McDonald et al. 2014). It is believed that optimizing intravenous hydration is a protective measure against developing contrast-induced AKI (Gupta et al. 2016), however, there are no recommendations for preventing AKI during PMT.

## Conclusion

Despite the fact that PMT plays an important role in endovascular treatment of both venous and arterial thrombosis, there are potential complications such as developing dialysis-requiring AKI. Until there is sufficient evidence and recommendation for the prevention of AKI in this setting, we believe that being aware of the risks and with close monitoring of patient, one might reduce the risk of severe AKI and its consequences.

## Data Availability

The datasets used and/or analysed during the current study are available from the corresponding author on reasonable request.

## Abbreviations

AKI: Acute kidney injury
CDT: Catheter directed thrombolysis
DSA: Digital subtraction angiography
MDT: Multidisciplinary team
PMT: Percutaneous mechanical thrombectomy

## References

[CR1] Arslan B, Turba UC, Matsumoto AH (2007). Acute renal failure associated with percutaneous mechanical thrombectomy for iliocaval venous thrombosis. Semin Intervent Radiol.

[CR2] Bedi PA, Daniel & Reichman, Aaron & Chou, Shyan-Yih & Reiser, Ira (2018) Acute kidney injury requiring renal replacement therapy due to severe hemolysis after mechanical thrombectomy | Request PDF. Case Rep in Intern Med 3(4):87–90. http://www.sciedupress.com/journal/index.php/crim/article/view/10269/6367

[CR3] Dukkipati R, Yang EH, Adler S, Vintch J (2009). Acute kidney injury caused by intravascular hemolysis after mechanical thrombectomy. Nat Clin Pract Nephrol.

[CR4] Escobar GA, Burks D, Abate MR, Faramawi MF, Ali AT, Lyons LC (2017). Risk of acute kidney injury after percutaneous Pharmacomechanical Thrombectomy using AngioJet in venous and arterial thrombosis. Ann Vasc Surg.

[CR5] Gupta R, Moza A, Cooper CJ (2016). Intravenous Hydration and Contrast-Induced Acute Kidney Injury: Too Much of a Good Thing?. J Am Heart Assoc.

[CR6] Mathews JC, Pillai U, Lacasse A (2011). Prolonged renal failure post-percutaneous mechanical thrombectomy. NDT Plus.

[CR7] McDonald JS, McDonald RJ, Carter RE, Katzberg RW, Kallmes DF, Williamson EE (2014). Risk of intravenous contrast material-mediated acute kidney injury: a propensity score-matched study stratified by baseline-estimated glomerular filtration rate. Radiology..

[CR8] Morrow KL, Kim AH, Plato SA, II, Shevitz AJ, Goldstone J, Baele H, et al (2017) Increased risk of renal dysfunction with percutaneous mechanical thrombectomy compared with catheter-directed thrombolysis. J Vasc Surg 65(5):1460–1466 10.1016/j.jvs.2016.09.047 27876521

[CR9] Registry of AngioJet Use in the Peripheral Vascular System - Study Results - ClinicalTrials.gov. 2018

[CR10] Schrijver AM, de Vries JP, van den Heuvel DA, Moll FL (2016). Long-term outcomes of catheter-directed thrombolysis for acute lower extremity occlusions of native arteries and prosthetic bypass grafts. Ann Vasc Surg.

[CR11] Sebastian H, Tam N, Karumathil M (2018). AngioJetTM rheolytic thrombectomy induced intravascular haemolysis leading to acute kidney injury requiring dialysis. J Clin Nephrol.

